# Down-regulation of the pro-apoptotic XIAP associated factor-1 (XAF1) during progression of clear-cell renal cancer

**DOI:** 10.1186/1471-2407-9-276

**Published:** 2009-08-08

**Authors:** Carsten Kempkensteffen, Florian Rudolf Fritzsche, Manfred Johannsen, Steffen Weikert, Stefan Hinz, Manfred Dietel, Marc-Oliver Riener, Holger Moch, Klaus Jung, Hans Krause, Kurt Miller, Glen Kristiansen

**Affiliations:** 1 Department of Urology, Charité – Universitätsmedizin Berlin, Charitéplatz 1, 10117 Berlin, Germany; 2 Institute of Surgical Pathology, UniversitätsSpital Zürich, Schmelzbergstr. 12, 8091 Zurich, Switzerland; 3 Institute of Pathology, Charité – Universitätsmedizin Berlin, Charitéplatz 1, 10117 Berlin, Germany; 4 Berlin Institute for Urologic Research, Berlin, Germany

## Abstract

**Background:**

Decreased expression of the interferon-stimulated, putative tumour suppressor gene XAF1 has been shown to play a role during the onset, progression and treatment failure in various malignancies. However, little is yet known about its potential implication in the tumour biology of clear-cell renal cell cancer (ccRCC).

**Methods:**

This study assessed the expression of XAF1 protein in tumour tissue obtained from 291 ccRCC patients and 68 normal renal tissue samples, utilizing immunohistochemistry on a tissue-micro-array. XAF1 expression was correlated to clinico-pathological tumour features and prognosis.

**Results:**

Nuclear XAF1 expression was commonly detected in normal renal- (94.1%) and ccRCC (91.8%) samples, without significant differences of expression levels. Low XAF1 expression in ccRCC tissue, however, was associated with progression of tumour stage (p = 0.040) and grade (p < 0.001). Low XAF1 tumour levels were also prognostic of significantly shortened overall survival times in univariate analysis (p = 0.018), but did not provide independent prognostic information.

**Conclusion:**

These data suggest down-regulation of XAF1 expression to be implicated in ccRCC progression and implies that its re-induction may provide a therapeutic approach. Although the prognostic value of XAF1 in ccRCC appears to be limited, its predictive value remains to be determined, especially in patients with metastatic disease undergoing novel combination therapies of targeted agents with Interferon-alpha.

## Background

Renal cell carcinoma (RCC) of the clear-cell type accounts for 3% of all adult malignancies and exhibits the highest cancer-related mortality among urological cancer entities [1]. Although the majority of patients (70%) present with localized RCC at the time of diagnosis, approximately 40% progress to metastatic disease following tumour surgery [2,3]. Once metastases are diagnosed, median survival rates drop to less than one year, mainly due to the fact that RCC is largely refractory to conventional cytotoxic therapies [2,4]. The investigation of molecular parameters involved in the development, metastatic spreading and treatment resistance of RCC may help to develop new therapeutic strategies as well as to identify molecular makers that better characterize the aggressiveness of the individual tumour than standard clinico-pathological predictors [5-8].

The ability of neoplastic cells to evade apoptosis is known to play an essential role for the development, progression and treatment resistance of cancer [9,10]. X-linked inhibitor of apoptosis (XIAP) is the best characterized and most potent member of the inhibitor of apoptosis (IAP) family [11-13]. Its caspases-inhibitory activity accounts for the protective effect against several apoptotic triggers including irradiation and various anti-cancer drugs[14]. The pro-survival activity of XIAP can be reversed by IAP-antagonists such as the mitochondrial protein Smac/DIABLO (second mitochondria-derived activator of caspases/direct IAP-binding protein with low pI) [15,16] and the nuclear protein XAF1 [17,18]. XAF1 has been identified as an interferon (IFN)-inducible tumour suppressor gene, which's expression sensitizes cancer cells to several apoptotic stimuli [18,19]. The pro-apoptotic effects of XAF1 may be mediated by direct sequestration of XIAP from the cytosol to the nucleus, thus antagonizing the inhibition of caspases [18]. More recently, XIAP-independent pathways of apoptosis-sensitization by XAF1 have been identified, e.g. the promotion of cytochrome c release, the prolonged activation of p53 protein and its target gene expression as well as the degradation of the IAP-family member survivin [20-22]. XAF1 is ubiquitously expressed in normal human tissues, but at comparably low or undetectable levels in numerous cancer cell lines with high XIAP expression on the other hand [17,18]. These data suggest that either down-regulation of XAF1 or up-regulation of XIAP expression may promote the survival of tumour cells [17,23]. In deed, over-expression of XIAP protein has been related to RCC progression and an unfavourable outcome in RCC patients [24,25]. Conversely, transcriptional down-regulation of XAF1 expression has been reported to occur in RCC [21] and low XAF1 mRNA tumour levels have also been linked to impaired prognosis in RCC patients [26]. However, to further clarify the potential relevance of XAF1 for the development and progression of ccRCC, it is essential to investigate whether those mRNA-based findings translate to the protein level. This study was done to examine XAF1 protein expression in a large cohort of ccRCC patients and to investigate the impact of XAF1 expression on clinico-pathological parameters and outcome.

## Methods

### Collection of samples

Two-hundred-ninety-one patients (197 men, 94 women) diagnosed with ccRCC at the Institute of Surgical Pathology, University Hospital Zurich and the Institute of Pathology, Charité – University Medicine Berlin between 1993 and 2005 were included in the present investigation. The study has been approved by the Charité University Ethics Commitee and the ethics committee of the University of Zurich. Non-neoplastic tissue samples of 68 patients from Berlin (55 men, 13 women) were subsequently included. Histological diagnosis was established according to the guidelines of the World Health Organization. All cases were selected according to tissue availability and were not stratified for any known preoperative or pathological prognostic factor.

### Tissue Micro Array construction

A tissue-micro-array (TMA) was constructed as previously described [27]. Briefly, suitable areas for tissue retrieval were marked on standard haematoxylin/eosin (H/E) sections, punched out of the paraffin block and inserted into a recipient block. The tissue arrayer was purchased from Beecher Instruments (Woodland, USA). A punch diameter of 0.6 mm was applied. Each tumour was represented by two (Berlin) or one (Zurich) tissue core, respectively.

### Immunohistochemistry

Formalin fixed paraffin embedded tissue was freshly cut (3 μm). The sections were mounted on superfrost slides (Menzel Gläser, Braunschweig, Germany), dewaxed with xylene and gradually hydrated. For immunohistochemical staining, we used the Vantana Benchmark platform with a standard antigen retrieval program (pressure cooking). The primary polyclonal XAF1 antibody (Imgenex, CA, USA, Catalog No IMG-379) was used in a dilution of 1:200. UltraVIEW™ was used as detection system with Diaminobenzidin (Sigma-Aldrich, Munich, Germany) serving as chromogen. Thereafter, the slides were briefly counterstained with haematoxylin and aquaeously mounted.

### Evaluation of the immunohistochemical stainings

Two genitourinary pathologists evaluated the TMA on a multi-headed microscope. The nuclear staining quantity was evaluated with percentages of cells stained in analogy to routine Ki-67 immunostainings in categories of 10% intervals. The staining intensity was evaluated in a four tire system from 0 (no staining) to 3 (strong staining). Printouts of all four staining intensities were at hand as a reference during the evaluation. An immunoreactive score (IRS) was computed by multiplying staining intensity and quantity. We used the median IRS value as cut-off for dichotomizing patients into subgroups with low and high XAF1 expression. Subsequently, the XAF1 expression was evaluated in 68 normal renal tissue samples. A complementary subset of 50 papillary and 17 chromophobe RCC was immunohistochemically analysed.

### Statistical analysis

Statistical analysis was performed using SPSS, version 15.0. Fisher's exact tests and χ^2^-tests were applied to assess statistical associations. Univariate survival analysis was carried out according to the Kaplan-Meier method and differences in overall survival were calculated using the log-rank test. Multivariable analyses were performed constructing Cox proportional hazards models. All p values were two-tailed, and those < 0.05 were considered statistically significant.

## Results

### XAF1 expression in normal renal tissue and clear-cell renal cell carcinomas

**Figure 1:**
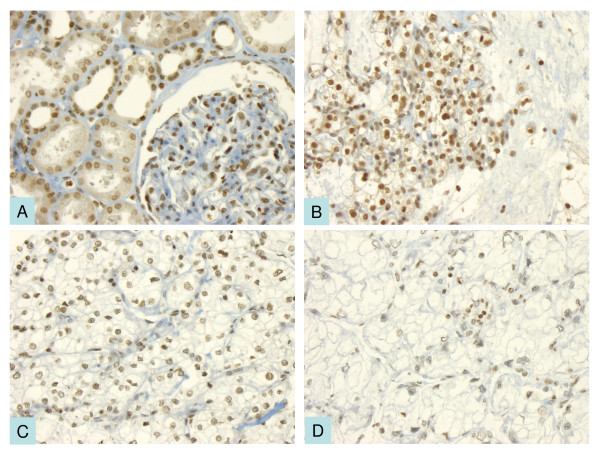
**XAF1 immunohistochemistry**. **A **Normal renal tissue with prominent nuclear XAF1 expression in the tubuli and glomerular cells. **B-D **XAF1 expression in clear cell carcinomas with high (**B**), intermediate (**C**) and low (**D**) immunoreactive scores respectively.

XAF1 was expressed in 64 of 68 benign renal samples (94.1%), with a nuclear expression pattern. Nuclear XAF1 expression was consistently present in the epithelia of tubules showing predominance in distal tubules over proximal tubules (Figure 1A). Furthermore, mesangial cells of glomeruli were consistently positive for XAF1. The median IRS calculated for the normal renal samples was 40 (range of 0 – 180).

XAF1 expression was detected in 267 of 291 ccRCC samples (91.8%), likewise showing a nuclear expression pattern. Although the intensity of XAF1 expression in tumour tissue seemed to be more variable than in benign renal parenchyma, the median IRS of 50 (range of 0 – 180) did not differ statistically significant from that observed in normal renal tissue. Figure 1B–C displays the different staining patterns of XAF1 in ccRCC. Both, benign and malignant samples lacking nuclear XAF1 expression also did not display cytoplamatic staining but were completely negative for this marker. A complementary analysis of 50 papillary and 17 chromophobe RCC mirrored the above described findings concerning staining pattern and quantity with negativity rates of 8% and 17.6% respectively. The median IRS value for these tumour subtypes was 35 and 20. These complementary data were not included in the further analyses.

### Association of XAF1 with clinico-pathological tumour features and outcome

The clinico-pathological characteristics of the study population are depicted in Table 1. Follow-up data was available for all patients included in the study, with a median follow-up time of 35 months (range of 1–139) months. 103 of 291 patients (35.4%) had died of ccRCC during follow-up, but 50 patients had metastases at time of surgery already. To correlate XAF1 expression to clinico-pathological tumour characteristics and prognosis, patients were dichotomized into subgroups with high and low XAF1 expression using the median IRS in ccRCC as a cut-off.

**Table 1:** Associations (χ^2^- and Fisher exact tests) between nuclear XAF1 protein expression levels and clinico-pathological parameters in clear-cell RCC

All cases		Total (%)	XAF1 low (%)	XAF1 high (%)	p-value
		291 (100)	152 (52.2)	139 (47.8)	
**Gender**					0.212
	Men	197 (67.7)	108 (54.8)	89 (45.2)	
	Women	94 (32.3)	44 (46.8)	50 (53.2)	
**Age**					0.101
	≤64	142 (48.8)	67 (47.2)	75 (52.8)	
	>64	149 (51.2)	85 (57.0)	64 (43.0)	
**pT-status**					0.040
	pT1/2	145 (49.8)	67 (46.2)	78 (53.8)	
	pT3/4	146 (50.2)	85 (58.2)	61 (41.8)	
**Fuhrman grade**					<0.001
	G 1/2	129 (44.3)	50 (38.8)	79 (61.2)	
	G 3/4	162 (55.7)	102 (63.0)	60 (37.0)	
**Metastases**					0.276
	M0/x	241 (82.8)	122 (50.6)	119 (49.4)	
	M1	50 (17.2)	30 (60.0)	20 (40.0)	

Low XAF1 expression was significantly related to tumour progression. The proportion of patients with low XAF1 tumour levels was significantly higher in the subgroup of ccRCCs with T1/T2 disease compared to T3/T4 disease. (p = 0.040). Moreover, patients with low XAF1 tumour levels were more likely to suffer from poorly or undifferentiated ccRCCs, compared to those with high expression levels (p < 0.001). No significant associations were observed between XAF1 expression and the metastatic status at time of surgery or patients' age and gender (Table 1).

**Figure 2:**
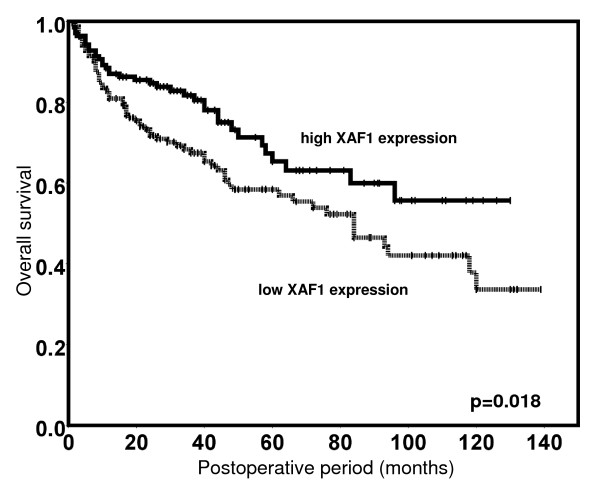
**Kaplan-Meier estimates of cumulative overall survival in clear-cell RCC patients according to the nuclear expression levels (high versus low) of XAF1**. Patients with low nuclear XAF1 expression levels (dotted line) display significantly shortened overall survival times compared to those with high nuclear XAF1 expression levels (bold line).

In Kaplan-Meier analysis, low XAF1 expression was significantly associated with a shortened overall survival (Figure 2). The mean overall survival was 91 months (CI 95%: 80 – 101) for patients with high XAF1 expression and 79 months (CI 95%: 68 – 89) for those with low XAF1 tumour levels (p = 0.018). In analogous univariate survival analyses, standard pathological parameters for outcome prediction of RCC patients also reached statistical significance (Table 2). In multivariable Cox regression analysis XAF1 expression did not provide independent prognostic information, whereas the conventional prognosticators, e.g. tumour stage, grade and the metastatic status at time of surgery, maintained their prognostic value (Table 3).

**Table 2:** Univariate survival analysis (Kaplan-Meier) and log rank test

Characteristic		No. of cases	No. of events	Two-year survival rate (± SE) in %	p-value
**XAF1 expression **		291	103		
	Low	152	66	71.8 ± 3.7	0.018
	High	139	37	84.7 ± 3.1	
**PT-Status**					< 0.001
	pT1/2	145	28	92.3 ± 2.2	
	pT3/4	146	75	63.7 ± 4.0	
**Fuhrman grade**					< 0.001
	G 1/2	129	25	88.1 ± 2.9	
	G 3/4	162	78	69.4 ± 3.7	
**Metastases**					< 0.001
	M0/x	241	68	84.8 ± 2.3	
	M1	50	35	43.7 ± 7.3	

Survival times of patients with clear-cell RCC according to clinico-pathological characteristics and nuclear XAF1 protein expression levels.

**Table 3:** Multivariable survival analysis (Cox regression)

Characteristic	Relative Risk	95% confidence intervall	p-value
**XAF1 expression**	1.200	0.792–1.820	0.390
**pT-status**	2.481	1.571–3.918	< 0.001
**Fuhrman grade**	1.736	1.083–2.784	0.022
**Metastases**	3.675	2.367–5.707	< 0.001

Characteristics are dichotomized according to Table 1.

## Discussion

This is the first study to demonstrate that low XAF1 protein expression is related to tumour progression in patients with ccRCCs. Low XAF1 tumour levels are also associated with a shortened overall survival of ccRCC patients but do not provide independent prognostic information when adjusting for standard pathological parameters for outcome prediction of renal cancer.

XAF1 mRNA is expressed at low or undetectable levels in most cancer cell lines, and transcriptional down-regulation in tumour as opposed to corresponding normal tissues has been shown to occur at different frequencies in gastric adenocarcinomas, colorectal cancer, urothelial carcinomas, malignant melanomas and also clear-cell renal cell carcinomas [17,18,21,28-30]. Although these data suggest a functional relevance of XAF1 down-regulation in the process of malignant transformation, little is known about protein expression of XAF1 and one cannot assume that XAF1 mRNA levels regularly translate to the protein level. In this context it has recently been shown that IFN-treatment of various tumour cell lines lead to a strong up-regulation of XAF1 mRNA but did not affect protein expression [19].

In our study we detect XAF1 only as a nuclear staining in both normal and malignant renal tissue. Interestingly, Augello et al. found nuclear and cytoplasmic stainings for XAF1 in neoplastic as well as non-neoplastic hepatic lesions [31]. Zhang et al. and Sakemi et al. detected XAF1 only in the cytoplasm of normal and neoplastic hepatic tissues [32,33]. Meanwhile, Shibata et al. detected XAF1 likewise only in the nucleus of basal cells of the gastric mucosa and of gastric carcinomas [34]. This might hint at different functions of XAF1 in different histological types of tissue but it should also be mentioned that all these studies, except ours and that of Sakemi et al., used different XAF1 antibodies.

In the current study we demonstrate that the median XAF1 protein expression level in ccRCC tissue does not differ significantly from that observed in normal renal parenchyma. These data clearly point out that XAF1 expression is not altered during the process of ccRCC development and therefore suggest down-regulation of XAF1 to be irrelevant for ccRCC tumorigenesis. However, low XAF1 tumour levels may provide survival advantages of neoplastic cells in the presence of various apoptotic triggers and may thus still have a role for tumour progression [18,35]. Indeed, we found low XAF1 protein expression in ccRCC to be associated with progression of tumour stage and grade. These findings are in line with the results of other studies, reporting XAF1 expression levels to inversely correlate with tumour progression in gastric, bladder and colorectal cancer [21,30,35], but again, these investigations mainly focused on XAF1 mRNA expression and thus hamper a direct comparison to our data. Moreover, based on the study design, it remains to some point speculative whether down-regulation of XAF1 actually plays a role for ccRCC progression or whether the observed correlations only just constitute associated epiphenomena. Nevertheless, a correlation between low XAF1 protein expression and progression of tumour stage and grade has also been reported in hepatocellular carcinomas, supporting a possible functional relevance of XAF1 down-regulation for the progression of various malignancies including ccRCCs [32].

Corresponding to the observed associations of low XAF1 protein expression with advanced tumour stages and grades, we found low XAF1 tumour levels to predict an impaired prognosis in univariate, but not in multivariable analyses. In our previous, real-time RT-PCR based investigation, XAF1 mRNA expression levels did not relate to histopathological parameters, but independently predicted an unfavourable clinical course of RCC patients [26]. So basically both finding are absolutely in line in terms of the adverse effects and associations of XAF1 down-regulation in ccRCC. The described differences could be due to variations in the size of the cohort, but might also indicate that XAF1 mRNA levels do not directly relate to the respective protein levels. Such differences have already been published by Leamen et al. for XAF1 in melanoma cell lines [19].

By analogy with down-regulation of XAF1 protein expression, up-regulation of its antagonist XIAP has also been shown to be associated with RCC progression and poor prognosis of RCC patients [24,25]. Since the decision whether a cell undergoes apoptosis depends on the balance between pro- and anti-apoptotic factors like XAF1 and XIAP [18], the concurrent evaluation of these parameters in RCC, represented through the XIAP to XAF ratio, may help to improve the prognostic value of these parameters compared to separate analyses. The feasibility of this concept has recently been proven in an immunohistochemical investigation on the prognostic effect of XIAP and XAF1 in gastric cancer [34].

Despite its uncertain value as a prognostic parameter, decreased expression or inactivation of XAF1 has been demonstrated to confer protection from various apoptosis-inducing triggers including 5-fluorouracil, etoposide and γ-irradiation [18,35]. Consequently, an abnormal reduction of XAF1 expression, predominantly observed in locally advanced and poorly differentiated RCCs, may contribute to their extraordinary resistance to conventional chemo- and radiotherapy [4]. Furthermore, XAF1 has been identified as an interferon-stimulated gene (ISG), which's expression is up-regulated by the exposure to IFN-α and IFN-β, resulting in a sensitization of various tumour cell lines to tumour necrosis factor-related apoptosis inducing ligand (TRAIL)-induced apoptosis [19,36]. Up-regulation of XAF1 protein expression augmented IFN-induced apoptosis, whereas down-regulation conferred protection from IFN-induced apoptosis in the renal cell carcinoma cell line ACHN [37]. This indicates XAF1 not only to constitute an ISG, but also to mediate the anti-tumour effects of IFN. Therefore, one can speculate that both, the failure of IFN to induce XAF1 expression in IFN-resistant tumour cells as well as an abnormal reduction of XAF1 protein expression, may contribute to the poor response rates of ccRCC patients to IFN-based immunotherapy [38,39]. Although single-agent IFN-α therapy is meanwhile obsolete for metastatic ccRCC patients, this aspect deserves further investigation, since its combination with targeted agents like Bevacizumab is an established therapeutic option for first-line treatment [40]. Considering that XAF1 has recently been reported to predict treatment response of bladder cancer patients to chemotherapy [41], it appears also reasonable to evaluate its predictive value in ccRCC patients as well. Finally, XAF1 itself may potentially provide a therapeutic agent, as its induction by adenoviral delivery recently proved to be promising strategy for the treatment of cancer [42].

## Conclusion

In conclusion, low XAF1 protein levels in ccRCCs were related to poor tumour differentiation, advanced tumour stages and unfavourable outcome. Although XAF1 did not provide independent prognostic information, these observations fit well with the pro-apoptotic activities of XAF1 and suggest its down-regulation to play a role during the process of ccRCC progression. Further studies aiming to evaluate the predictive value and therapeutic potential of XAF1 in ccRCCs are warranted.

## Competing interests

The authors declare that they have no competing interests.

## Authors' contributions

CK and FRF conceived the study, performed immunohistological and statistical analyses and wrote the manuscript. MJ, SW, MOR and SH contributed to the manuscript preparation. KJ and HK provided samples and contributed to the study design. KM critically revised the manuscript. MD and HM provided samples and clinico-pathological data. GK coordinated the study, performed immunohistological and statistical analyses, supplied administrative support and revised the manuscript. All authors read and approved the final manuscript.

## Pre-publication history

The pre-publication history for this paper can be accessed here:

http://www.biomedcentral.com/1471-2407/9/276/prepub
